# The dynamic use of a balanced scorecard in an Italian public hospital

**DOI:** 10.1002/hpm.3440

**Published:** 2022-02-20

**Authors:** Gaia Bassani, Chiara Leardini, Bettina Campedelli, Sara Moggi

**Affiliations:** ^1^ Department of Management University of Bergamo Bergamo Italy; ^2^ Department of Business Administration University of Verona Verona Italy

**Keywords:** balanced scorecard, health, performance management, performance measurement, use

## Abstract

**Purpose:**

This paper aims to analyse the dynamic use of the balanced scorecard (BSC) in an Italian public hospital.

**Design/Methodology/Approach:**

A longitudinal case study was conducted at an Italian public teaching hospital over a period of 5 years. The emergence of dynamic use of BSC was traced over a different combination of social, political, economic and organizational realities. A deeper understanding of these realities requires the adoption of a holistic approach to BSC use. Henri's types of system use (i.e., monitoring, attention focussing, strategic decision‐making and legitimizing) frame this approach in a more concrete manner.

**Findings:**

This study adds to the debate on whether BSC is used for aspects other than monitoring in public contexts. The case study offers the first example of a legitimizing use of the system and a first longitudinal case study that traces a dynamic use of BSC: the use evolves from monitoring and attention focussing to monitoring and legitimization. Norms, political parties and top managers play a determining role in this process.

**Originality/Value:**

Through a longitudinal approach, this study presents how BSC can be a dynamic tool steered by legitimacy pressures. The longitudinal study explores how social, political, economic and organizational context shape the implementation and the revision of BSC affecting the use of the tool by top managers. The browse of this dynamism is supported by Henri's type of use along with an in‐depth analysis of the BSC literature evolution in terms of its ‘static, dynamic and expected’ use.

## Highlights

- Performance management systems, such as balanced scorecard (BSC), should be understood in a holistic view, namely taking into account the interdependency between the design, the implementation, and the use of the systems and the interdependency between different systems operating at the same time in the same organization.
- How managers use BSC in hospitals changes over time.
- At an organizational level, a BSC could provide justification of actions and decisions as well as monitoring results, supporting strategic decision‐making processes and focussing attention on priorities.
- BSC is a pivotal lever that can be governed by hospital managers as a strategic management tool for facing legitimacy pressures.

## 1 INTRODUCTION

Although the meaning of the balanced scorecard (BSC) in healthcare organizations is ambiguous because of the type of control structure,^1^ there is a growing diffusion of BSC in the public health sector,^1^, ^2^, ^3^, ^4^, ^5^ in particular for monitoring hospital performance.^6^

Despite this increasing interest, the extant literature lacks a holistic approach to understanding the use of BSC. Most studies about BSC in healthcare contexts appear to be anchored to the design and implementation phases of a system's development, leaving little space for a description of the system at work. Cases^1^, ^2^, ^6^, ^7^, ^8^, ^9^ are numerous and cover different healthcare organizations and countries.

In all these cases, the authors describe a situation of use that does not change during the period of observation. Henri^10^ (97) describes as static those studies that ‘do not incorporate the evolution of a performance measurement system […] over time’. These studies deal predominantly with the expected use of BSC because their investigations cease before the system works properly. When the analysis focuses on the initial phases of a performance management system's (PMS) development, authors can comment only on what may be expected.^11^ This is the case of numerous researches.^2^, ^12^, ^13^ These findings reopen the debate on how BSC serves top managers in public^14^ and health domains.^1^, ^3^, ^4^

In terms of providing a dynamic image of the development and use of the system, a longitudinal investigation could be helpful. A scan of the literature provides few examples of longitudinal case studies on BSC. Funck^7^ and Aidemark^1^ describe evidence of Swedish healthcare networks, arguing that BSC provides a framework to make different stakeholders' interests visible and promotes communication based on shared performance results. In the same context, Aidemark and Funck^15^ find that BSC developed in a Swedish medical clinic is useful to measure the results and behaviours of workers at a ward level. These studies present the design, implementation and use of BSC through the years but they do not reveal the change in the system itself or in the use of the system by top managers. An in‐depth literature review reveals scant literature on the BSC dynamism in terms of its attributes and uses.

Considering this gap, this study aims to deepen the concept of the use of BSC in public health considering how social, political, economic and organizational context shape the implementation and the revision of BSC affecting the use of the tool by top managers. In doing so, the study introduces the distinction between ‘static, dynamic and expected’ uses of BSC. In addition, the paper examines the use of BSC employing ‘Henri's^10^ types' regarding the nature of performance measurement systems' use. This framework goes beyond the debate on BSC as a performance management or performance measurement system. In fact, Henri^10^ addresses, among other uses, both reporting and decision‐making uses. Thus, the distinction between performance measurement and PMSs in this paper loses relevance.

For exploring the dynamism of BSC over time affecting the use of the tool by top managers, the case of an Italian public hospital has been considered. This case study has been explored considering a longitudinal approach in the data collection. This approach permitted an in‐depth analysis of the BSC evolution in features and uses over time considering the multiple stakeholders involved in its design and implementation.

The contribution of the paper is threefold. First, the study contributes to the scant literature that considers the BSC as a dynamic tool of performance management for improving legitimacy on the organization. Second, a theoretical contribution is presented, considering a dynamism in the model proposed by Henri.^10^ Third, the paper proposes a novel point of view that managers using BSC in a PMS should consider suggesting that, instead of a static tool, BSC can be shaped over time in accordance with legitimacy pressures.

In the next section, we develop a broad approach to PMSs based on an in‐depth literature review. Following this, we describe the research methodology and then analyse the dynamic use of BSC in our case hospital and the context in which it is embedded. The final sections discuss our interpretation of the case according to the theoretical framework and present our conclusions.

## 2 THE CONCEPTUAL FRAMEWORK REGARDING THE USE OF BALANCED SCORECARD IN HEALTH CARE CONTEXTS

A scan of the extant literature on BSC in health care reveals the resilience of the system^16^ in terms of the adaptation to new roles, contexts and actors. However, studies largely ignore the interdependency between the design, the implementation, and the use of BSC and the interdependency between different systems operating at the same time in the same organization.^17^, ^18^ Therefore, we argue that research on BSC would benefit from a holistic view of the key aspects that intervene at different moments during the process. This understanding promotes a continuous analysis on how to introduce and reshape BSC according to the different uses that top managers make of the system. Thus, the adoption of a holistic view reveals the dynamic use of BSC and the political, social and economic relationships that could contribute to this dynamism. In this sense, BSC can also become a valuable strategic management tool and support managers in their decisions.^19^, ^20^

This approach shares the same rationale as Ferreira and Otley's^17^ study, encouraging a broader view of PMS that captures the richness of issues and relationships implicated in PMS design and use. In the public sector, the politicized nature of decision‐making^3^, ^21^ is acknowledged, as is the fact that systems and activities rely on building consensus between top managers and politically elected bodies. The process of consensus building should take into account the long‐term view promoted by top managers and the short‐term emphasis traditionally dominating political regulation. Consequences on systems design and use are evident. First, in the case of coupling, the government regulation becomes intricately intertwined with organizational strategy, narrowing the strategic priorities and limiting the support that a PMS could give to top managers.^21^ Second, in terms of de‐coupling,^15^, ^19^, ^22^ organizations could compile more information than is required for decision‐making and could suffer from an excessive proliferation of performance indicators.^3^ In these dynamics, the top managers' style of leadership affects the subsequent coupling or de‐coupling behaviours.^21^

In these fluid contexts, the political, social and economic relationships are of extreme importance. Some authors emphasize how these relationships affect the nature of PMS use.^10^, ^23^ Henri,^10^ in particular, draws on various classifications of management and accounting information systems to define and operationalize the use of performance measurement systems by top managers. The analysis of these classifications reveals four types of use: **monitoring**, **attention focussing**, **strategic decision‐making** and **legitimization**.

According to Henri,^10^ top managers use performance measurement systems for **monitoring** when they should provide feedback and communicate with multiple stakeholders. Performance measures^24^, ^25^ populate reporting documents and top managers promote diagnostic control^26^ through the system. In addition, when top managers send priority messages throughout the organization, the system could be used more interactively,^26^ focussing attention on specific objectives and measures (**attention focussing use**). Further, performance measurement systems could be central to decision‐making processes, facilitating the choice of objectives and actions (**strategic decision‐making** use) or justifying decisions or actions (**legitimate use**). When performance measurement systems serve a strategic decision‐making use, the system is a collector of learning initiatives helping problem‐solving activities. Finally, performance measurement systems could form the basis for justifying past actions or decisions by seeking validation of current and future actions.

Our conceptual framework, thus, benefits from the adoption of a holistic approach and of the performance measurement systems uses defined by Henri.^10^ A broad view clarifies the trajectories of the different BSC uses made contextually and/or subsequently by the top managers. With trajectories we mean the interdependencies among the different systems operating in the organization and the political, social and economic relationships around it.

The fact that Henri's^10^ categorization comes from conceptual and empirical scouting of the systems in use in different contexts (i.e., private and public) is a plus in our discourse. It broadens the focus on uses beyond monitoring, which is the use most addressed by public management studies.^1^, ^3^, ^14^ We group the extant literature on BSC in health care according to Henri's^10^ four categories of use. As depicted in Table 1, the majority of previous studies describe BSC use and expected use in terms of monitoring. This result is similar to Northcott and Ma'amora Taulapapa's^14^ and Aidemark's^1^ concluding remarks. Further, BSC is used for attention focussing and strategic decision‐making. This is new evidence in these contexts. In support of the legitimization of objectives, no studies mention this potentiality of BSC use.

**TABLE 1:** Categorizing the extant literature according to Henri's^10^ types of system use

Streams and studies/Henri's^10^ uses of system	Monitoring	Attention focussing	Strategic decision‐making	Legitimization
Aidemark (2001)^1^	X			
Aidemark and Funck (2009)^15^	X			
Bobe et al. (2017)^16^	X	X		
Chan and Ho (2000)^8^	X			
Chang (2007)^9^	X			
Chang et al. (2002)^27^ ^,^ ^a^	*X*	*X*		
Chow‐Chua and Goh (2002)^28^ ^,^ ^a^	*X*			
Cleverley (2001)^29^ ^,^ ^a^	*X*	*X*		
Curtright et al. (2000)^30^ ^,^ ^a^	*X*			
Funck (2007)^7^	X	X		
Gordon et al. (1998)^12^	X	X	X	
Griffith et al. (2002)^31^ ^,^ ^a^	*X*			
Harber (1998)^32^	X	X	X	
Inamdar and Kaplan (2002)^13^	X	X	X	
MacStravic (1999)^33^ ^,^ ^a^	*X*		*X*	
Peters et al. (2007)^34^	X		X	
Pink (2001)^35^ ^,^ ^a^	*X*			
Rabbani et al. (2010)^36^ ^,^ ^a^	*X*			
Rimar and Garska (1999)^37^		X		
Sahney (1998)^38^ ^,^ ^a^	*X*			
Stewart and Bestor (2000)^39^		X		
Zelman (2003)^5^ ^,^ ^a^	*X*		*X*	

Abbreviation: BSC, balanced scorecard.

^a^ Studies on ‘expected use’ of BSC rather than use.

### 2.1 Methodology

#### 2.1.1 Research design/background

We present a longitudinal interpretive case study, carried out during 2010–2017 in an Italian teaching hospital, the Integrated University Hospital (IUH). IUH is a public organization that, together with the hospital and the directly linked structures, hospitalizes 60,000 people every year and provides jobs for 5000 people. In 2010, IUH adopted BSC to manage the merger of two organizations, the University Hospital and the Independent Hospital. Since 2010, IUH has undergone significant internal renovations and reorganization. A new surgical block was inaugurated in 2010, a health facility covering an area of more than 70,000 m^2^ that contains over 500 beds and 31 operating theatres. In 2017, the new Women's and Children's Hospital was inaugurated, hosting the units of paediatrics, obstetrics–gynaecology and their relative emergency rooms and intensive cares, paediatric surgery, paediatric oncohematology, and child neuropsychiatry.

IUH is located in Veneto, the third region in Italy for the GDP contribution, situated in the Northeast of the country. During these years, the Veneto Region faced the emergence of a new challenge induced mainly by the growth in life expectancy, the progressive ageing of the population and the increase in chronic degenerative diseases. Guaranteeing the appropriate care for all patients, this situation required a new and economically sustainable organizational model of social and health services. The new model is based on the hub and spoke network where public hospitals receive patients who required services at high intensity of care and the territorial health units manage the chronic patients who required services at low and medium intensity of care. As the central government grants greater independence to the regions in terms of health policy, Veneto uses a centralized/integrated model in which 80% of hospital beds are managed directly by the region through regional agencies. This is the highest percentage at the country level. Thus, the region shows a prevalence of public organizations that promote an integrated system of public and private healthcare services. Moreover, the President of the Veneto region, who has been in office since 2010, introduced a leading agency (i.e., Azienda Zero) in 2017, strengthening the management and monitoring of all the activities and financial trends of public and private healthcare organizations. Although the public administration already had a high level of centralization, this new decree continues this trend. All these changes stimulate a central strict management and monitoring of costs and financial flows.

Since the early stages of a benchmarking project, the President of Veneto has been promoting an interregional comparison of performances. In 2012, Veneto adhered to the project. At the central level, the advance of new public management initiatives was evident.^40^ The Italian government introduced the Decree Law no. 150/2009, which strongly stressed the role of the cycle of performance management in public organizations. This act promotes the rationale to manage and measure public organizations' results at both organizational and individual levels. The normative document does not specify the nature and form of performance tools. Thus, all the public entities' top managers directly establish objectives and resources based on their experience and on the strategic directions of the organizations they manage.

Further, in 2013 AGENAS, a national agency for regional healthcare services, introduced a national healthcare plan of results as mandated by the Ministry of Health. The aim of this tool was to monitor all the variables that affect the quality, efficiency and equity of healthcare activities. In 2015, a regional decree called for strict control on targets that were decided nationally. All organizations that reach the targets are subject to regional inspection. This trend continued in the yearly financial decrees of 2016 and 2017.

In this context, IUH was selected for deeper analysis for the present study because it has experimented with multiple changes over a relatively short time and has adapted to changes in the government's agenda.^41^ Moreover, researchers have a well‐established relationship with management accountants, top managers and some clinicians who work in IUH. Thus, both these aspects offer a promising opportunity to examine the organizational, managerial and strategic complexities associated with the adoption and use of BSC.

#### 2.1.2 Data collection

Case study approaches allow researchers to perceive the complexity of a single and unique case in its own context.^42^ The IUH longitudinal case permits the exploration of a BSC complexity and its evolution over time. A longitudinal case study provides an in‐depth view of the developmental phases of a particular phenomenon that resulted from several forces rooted in the research context.^43^ Data collection were carried out from 2013 to 2017 considering as main methods in‐depth interviews of key stakeholders involved in the BSC design and implementation (e.g., planning and control staff members, the division responsible, physicians). Nineteen interviews, a number of informal meetings and internal documents collections (e.g., planning and control dashboards, performance plans, BSC periodical reports), permitted the triangulation of sources and the achievement of the saturation level.^44^ Interviews followed a flexible framework that permitted to maintain the flow in the discussion and guided the discourse over three main issues: (i) general use of the BSC in IUH and its evolution over time; (ii) specific impacts of the BSC evolution on the interviewees daily work; (iii) main forces guiding the BSC changes. Questions slightly change according to the interviewees' role in the organization and the seniority (Table 2).

**TABLE 2:** Interview details

Interviewee role	Interview date	Code
Management Accountant	22/03/2013	I1
	10/11/2017	I8
Nurse Top Manager	10/11/2017	I9
Pharmacy Manager	14/06/2013	I5
	16/11/2017	I10
Management Accountant 1 who manages regional reports	22/03/2013	I2
	16/11/2017	I11
Management Accountant 2 who manages regional IT flows	22/03/2013	I3
	16/11/2017	I12
Quality Manager	14/06/2013	I6
	16/11/2017	I13
Integrated Department Manager (Physician 1)	16/11/2017	I14
Evaluation Committee Member	27/11/2017	I15
Management Accounting Manager	22/03/2013	I4
	27/11/2017	I16
HR Manager	11/12/2017	I17
Medical Director (Top Manager)	14/06/2013	I7
	12/01/2018	I19
Integrated Department Manager (Physician 2)	11/12/2017	I18

Interviews were transcribed and read many times reiteratively before being annotated extensively and the emerging codes or themes noted. The codes or themes coming from the respondents enrich the previous identification of codes derived from extant literature in accordance with the research objective. Thus, the data were filed according to the categories and codes selected.

### 2.2 Description of findings: The IUH case

To illustrate the dynamic use of BSC in IUH, we present our findings in two phases: the emergence of BSC (in 2010) and the revised BSC (in 2017). Our findings show that the developmental process and the different uses of BSC depend on political, economic, social, and organizational realities. Table 3 provides a screenshot of the realities in action.

**TABLE 3:** Political, economic, social and organizational realities in action

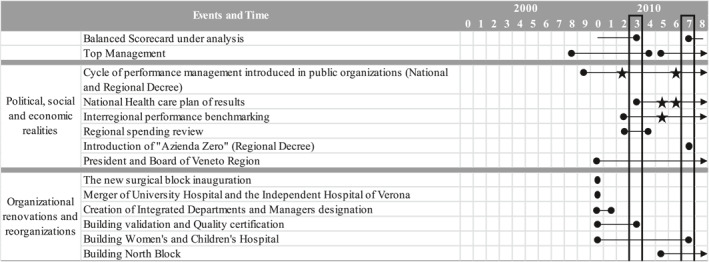

**FIGURE 1:**
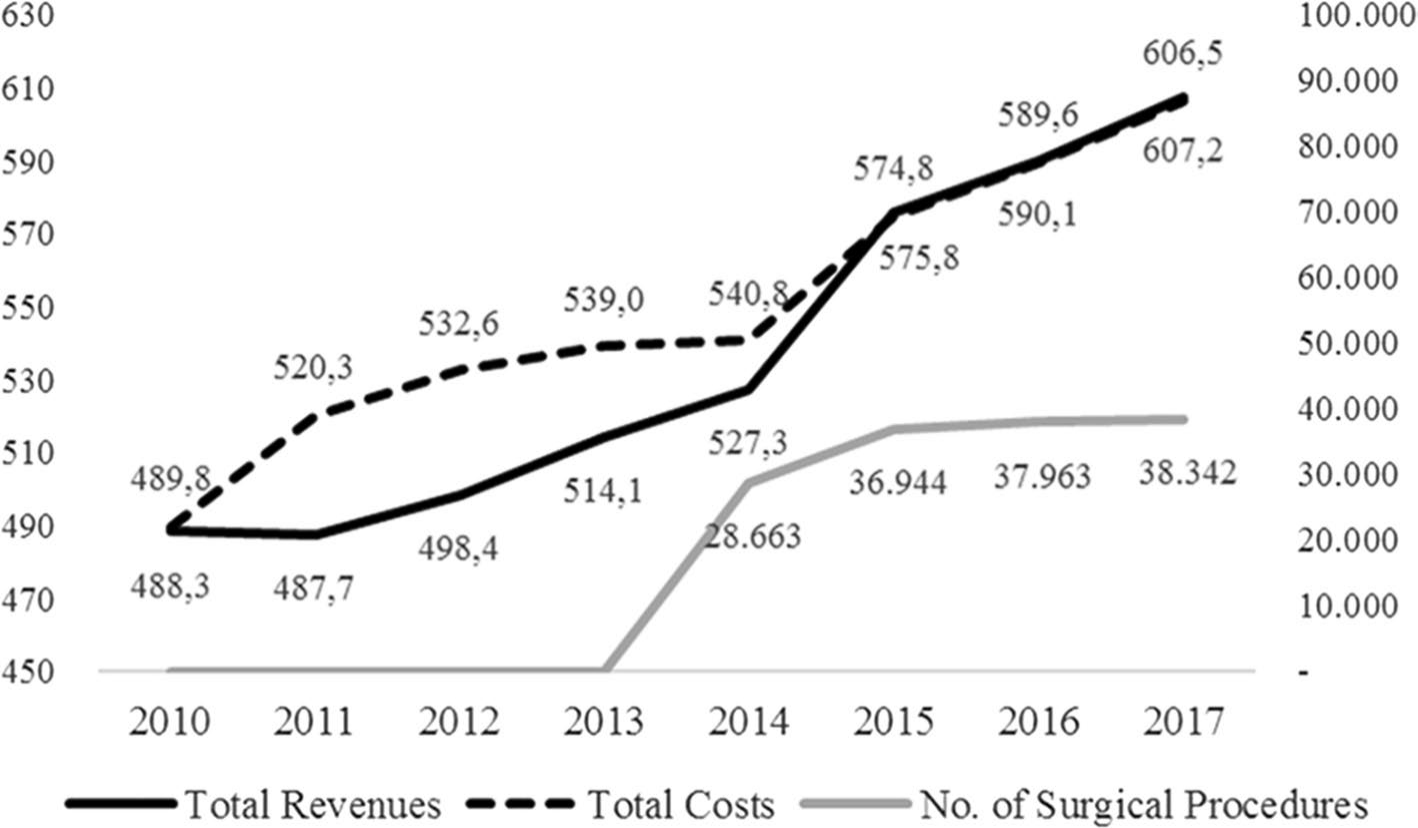
Integrated University Hospital performances

BSC significantly impacts financial performance. In 2015, IUH revenues exceed the amount of total costs. It was the first time in 18 years. Although the surgical procedures increase every year, the positive performance continues (Figure 1).

#### 2.2.1 The emergence of BSC in 2010

In 2010, after 2 years of their assignment, the General Manager and the Medical Director decide to introduce BSC as a reference system for the entire organization. Both managers, who had previous successful experience in BSC implementation, were struggling with the merger between the University Hospital and the Independent Hospital, the creation of integrated departments, and the finalization of the new surgical block.

In that period, the General Manager and the Medical Director were managing complex projects. They worked hard for the merger and the establishment of departments. In the meantime, they were building the surgical block. They had demanding objectives and responsibilities (I1).

The Medical Director leads the design and the implementation of BSC with the aim of executing the new mission of IUH. For him, all the organizational members should be familiar with the teaching hospital objectives and their daily activities should be executed according to these general directions.

The effort to implement BSC should be conceived in terms of a daily alignment of all the organizational members to hospital objectives (I7).

During the design process, the General Manager, the management accountants, the Pharmacy Manager and the Quality Manager map all the current objectives and information flows. They work together listing the priorities of the new organization (i.e., BSC perspectives) and connecting extant objectives and information to these priorities. Next, they simplify some objectives and targets for increasing the feasibility at the majority of internal areas and units. During this process, they guarantee the peculiarities of each unit in terms of activities specifying ad hoc targets and sub‐objectives. Though they prefer objectives and information flows already in use in the organization, in the case of new priorities and measurements, they involve other organizational members in the selection process. All the integrated departments' managers and other clinicians define objectives for measuring the results of their activities and the quality of the healthcare processes. Finally, each Unit Manager is asked to identify three objectives among those promoted by AGENAS for evaluating their daily activities. The choice to integrate the AGENAS objectives in the organizational BSC, instead of designing hospital‐made indicators, is properly justified by the Medical Director:

As we manage patient and citizen lives, they have the right to know our performance (I4).

Because the IUH top managers have received a strong mandate from the region to rationalize costs and lower the rate of hospitalization, the choice of some objectives is mandatory (I1, I4). Members do not perceive these strong mandatory requirements. In fact, the internal perception of spending review is mediated by the Medical Director's stimulus to innovate processes. Additionally, though surgical units managers are not impacted directly by the rate of hospitalization, they are invited to share the overall objective of lowering the number of invasive operations and increasing the accuracy of the activity. Working along with the entire design process strengthens the embeddedness of each actor in the IUH processes. This fact contributes to raising BSC as the point of reference for top managers and all organizational members.

The BSC was our bearings. We knew our priorities, our due activities, and our targets (I16).

As shown by internal documents provided by the Evaluation Committee, BSC presents 16 measures in four perspectives, namely activity, resources, professional quality and perceived quality. The first dimension, *activity*, measures the three main activities of IUH: health assistance (main objective of an independent hospital), research, and teaching (main functions of a teaching hospital). The Medical Director meditates the choice to insert the university and the hospital activities into the same perspective. The rationale is to communicate a united picture of the IUH activities. The second perspective, *resources*, reflects the financial aspects, taking into account costs, revenues, and efficiency. The third dimension, *professional quality*, captures the capacity of IUH to manage and perform health processes; this dimension involves process reengineering, medical results evaluation and projects.

The majority of clinicians and all the integrated departments' managers ask persistently to create this perspective. We satisfied them (I7).

The fourth perspective, *perceived quality*, monitors the satisfaction of both internal and external customers (BSC patient perspective) with a frequent survey. The items of the survey are identified in accordance with the measures of BSC.

The strong commitment of top managers is also evident in the implementation phase. The management accountants emphasize the top managers' interest in exploring the internal processes and involving all the clinicians, especially the newly established departments' managers (I1, I2, I4, I8, I11). The management accountants support the implementation and the spreading of the objectives and targets. Both the clarity and the high measurability of the objectives help members to focus on priorities. The use of BSC during the medical meeting permits that medical director share the strategy and organizational objectives, communicate and comment on the scorecard results with the department managers and unit managers.

When organizational units' results differ significantly, the Medical Director and the Management Accounting Manager guide a set of brainstorming for deepening the daily activities of each unit. The results stimulate a targets revision, distinguishing organizational units in clusters (I5).

A specific project was promoted for staff units (e.g., X‐ray laboratories), mapping their activities and identifying indicators that monitor the level of output for hospitalized patients and external services. BSC has improved management and decision‐making offering a common and integrated guideline to take decisions and increasing the understanding of how each dimension are part of a whole and could impact both financial performance and patient satisfaction. This BSC has strengthened the view on long‐term performance through a greater knowledge of the relationships among KPIs.

During the first years, BSC supports a path of convergence between the increasing activities of patient assistance, research and teaching and the will to reach the KPIs target. Usually, during the monitoring meetings, we discuss how the professional quality [a measure of the learning process] affects the total costs (I15).

In addition, this BSC provides for a continuous dialog between the top managers and the main actors (e.g., physicians and unit managers), prompting participative leadership that positively influences the use of the system. Indeed, one of the most effective advances of BSC is the negotiation of the budget, which leads to more awareness without the need to work through excessive red tape during this process phase. Top managers feel as though they are effectively managing the new integrated organization. They established the integrated departments, inaugurated the new surgical block, and obtained the building validation and quality certification. The IUH financial statements show a constant cost reduction and decrease in the rate of hospitalization.

In terms of perceived patient quality, BSC monitors the waiting times and also the number of problems patients have during hospitalization. The IUH internal staff consider these two targets according to the specific situation they were living. They would guarantee the full operation of the surgical block in the short run and they take into consideration the problems patients describe in a yearly survey.

#### 2.2.2 The revised BSC in 2017

**FIGURE 2:**
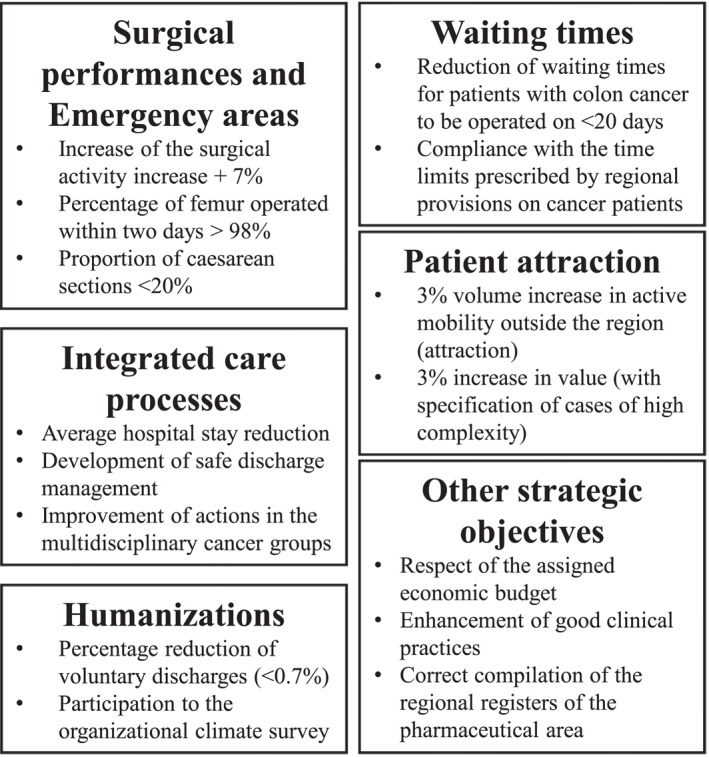
A choice of the balanced scorecard KPIs for the period 2017–2018

The management revise BSC every 2 years. Since 2015, the number of perspectives has increased, from four to seven. Table 4 summarizes the development of perspectives from 2011–2012 to 2017–2018. During these years, the Veneto region has identified points of weaknesses in the system (e.g., waiting times and efficiency of the emergency areas) and created ad hoc indicators (I16). Simultaneously, a new top management has taken office. For the last BSC revision (Figure 2), the current top management considered the same perspectives identified by the Veneto region as weaknesses. In terms of indicators, BSC 2017–2018 becomes relevant to external indicators.

The BSC 2017–2018 mixes those recommended by the Veneto region, by the national health care plan of results and by the interregional performance benchmarking (I8, I16).

**TABLE 4:** The development of BSC perspectives

BSC perspectives			
2011–2012	2013–2014	2015–2016	2017–2018
Activities (health assistance, research and teaching)	Activities (services functioning, waiting times, research and teaching)	Hospitalization and integrated care processes	Surgical performances
Resources use (costs, revenues and efficiency)	Resources use (service functioning, economic balance, times of mandatory information flows and reports)	Surgical and emergency areas	Patient attraction
Professional quality (process reengineering, medical results evaluation and projects)	Professional quality (process reengineering, medical results evaluation and projects)	Waiting times	Waiting times
Perceived quality (internal and external “customer” satisfaction)	Perceived quality (internal and external ‘customer’ satisfaction)	Patient attraction	Emergency areas and 118 service
‐	‐	Quality, safety and humanization	Integrated care processes
‐	‐	Economic balance	Humanization
‐	‐	Research and teaching	Other strategic objectives

Abbreviation: BSC, balanced scorecard.

The total number of indicators increased from 16 to 45, and just 10 indicators out of 45 have been chosen by IUH. When we asked how BSC is developed, some interviewees found it difficult to understand what system we were investigating.

Are you mentioning the budget? Or the cycle of performance management? (I9)

The current top management decides to adopt the same set of indicators for BSC and for the annual budget. The current Medical Director perceives BSC as a difficult system to manage and for communicating results to operating units (I16). She prefers to directly manage the information flows required by the region.

The only possibility we have is the definition of organizational mechanisms such as worktables. The region dictates the strategies and objectives. We must to be ready for answers (I19).

They eliminate those indicators that require a complex flow of inputs and analysis favouring the calculation of mandatory measures. Thus, the measures of professional quality that were a breath of fresh air in 2012 have been replaced by standardized measures required by the central government and the interregional benchmarking (I16). Physicians have not appreciated this choice.

Physicians have an appetite today. The new measures have evident limits connected to standardization (I13).

The current top management does not illustrate this choice to the physicians (I13). They directly inform management accountants of the new measures and priorities.

In 2017, the top‐down orientation is very strong. The current top managers abolish the routine meetings in which they present the strategic objectives, the BSC perspectives and measures. In that meeting, top managers discuss directly with physicians and unit managers about the strategic directions of IUH. Now, measures are decided and communicated top‐down (I13).

Though top managers seem focussed on regional requirements, the majority of staff managers and unit managers would be familiar with the overview of the hospital and its strategic directions (I8, I9, I13, I16). They would be more reactive management focussed on IUH needs instead of on compelling regional requirements. Moreover, they would create less interference during the choice of strategic investments and directions.

Last year, the region entrusted the total management of radiopharmaceuticals to a private hospital. We are all astonished! Some months before the region prevented us from buying a linear accelerator. It was detrimental for a competitive offer of services (I8).

As the region constantly asks for a prompt reply to false emergencies, the Management Accountant admits that it is difficult for top managers change their behaviour (I8).

It is acknowledged that in 2017, there is an excessive request for information by the region.

Controllers are more than workers are. I do not know if the region is able to manage all these data. They require rich details, lowering the significance of the information (I16), and they ask for redundant information flows (I12).

Moreover, the central government and other public administrations require additional details.

Usually, data required are the same asked by the region but differently aggregated (I16).

It seems a policy supervision (I12).

If you reach the target, you will be inside the system and vice versa (I12).

Since 2015, the Veneto region has suggested the use of ad hoc software for internal monitoring. They request the implementation of a standardized report for users. In 2016, the software was changed, and the region asked for a new implementation. All these systems seem reduce the possibility of directly managing the hospital.

There are no warnings that signal an inefficient situation of something similar. The region is interested in implementing standardized reports that are not at its disposal, but of which it knows all the information (I8).

In the near future, the region will probably implement a centralized management accounting and control system based on Azienda Zero (I8).

The regional supervisors give quarterly feedback and there are frequent inspections at the hospital.

When they announce their visit, the top managers prepare themselves one week in advance. It is an inspection, not a friendly visit and they want defence against accusations. The budget and the related information flows serve this purpose (I16).

In 2017, BSC guided the entire structure towards achieving regional objectives. It can be assumed that the BSC has been employed for managing and monitoring short‐term performance adapting the BSC objectives to KPIs requested at a regional level. Thus, BSC lacks attention on some organizational objectives such as teaching, research, and the quality of patient cares [client perspective] and loses the function of increasing internal collaboration and communication. In this way, the BSC loses its strategic function^45^ since it is driven by regional targets instead of IUH goals.

Finally, the Medical Director introduces weekly worktables during which the department managers, physicians and staff managers check the results of each indicator, comparing the result with the target imposed by the region (I16). In the beginning, the physicians think worktables are a means to share needs and problems with the top managers. However, after some meetings they understand that the only intention was to monitor their results and actions.

On the contrary, worktables appear as an unnecessary expansion of the power of control. Thus, physicians perceived worktables as a lack of trust (I9).

## 3 DISCUSSION OF FINDINGS

The finding highlights that the BSC use evolves from an emphasis on the system that helps top manager to set and communicate their priorities to a system used as a monitoring system and support legitimizing purposes. In this section, we discuss how the top managers use BSC within the public hospital. Henri's^10^ types of use are employed for enhancing our understanding of this matter in terms of BSC dynamism.

The 2017 BSC is a completely different system from that of 2013. According to other studies,^7^, ^8^, ^23^, ^28^, ^39^ the introduction of BSC is a voluntary practice promoted by the hospital's top managers. At the outset, BSC appears to be a proactive response to external forces including financial pressure, regulatory reporting^13^ and internal challenging objectives that top managers were managing. Though the system is no longer recognizable, it was still used in 2017. In that year, the top managers revised all the perspectives and measures according to the annual budget and the cycle of performance management.

### 3.1 Attention focussing use of BSC

The increase in perspectives and measures automatically decreases the attention‐focussing role of BSC over time. In terms of attention focussing, Henri^10^ refers to the interactive use of the system^26^ that helps top managers and users to focus on organizational priorities. Previous studies in which BSC is externally mandated explain the attention focussing with an emphasis on the strategic alignment between national and organizational objectives and measures.^16^, ^27^ In these situations, the top‐down approach followed in the translation process could disorient the internal managers and executives. As suggested by our findings, this is not usually the case when the introduction of BSC is a voluntary initiative. Being voluntary, the system helped top managers to set and communicate their priorities,^12^, ^13^, ^29^, ^39^ keeping managers focussed on critical areas.^29^

### 3.2 Monitoring use of BSC

In terms of monitoring, the two IUH top management teams clearly expressed the desire to obtain feedback, which was collected in reporting documents. This essential feedback satisfies the routine requests from the region and the other stakeholders (e.g., state and internal physicians). Further, the data are a useful means of communicating inside and outside the organization. Physicians, internal political parties and staff managers adapt their own language with the standard set of perspectives and measures adopted. The use of BSC for monitoring is well acknowledged across the literature.^1^, ^3^, ^14^ Henri^10^ identifies the monitoring type of use when top managers provide feedback and communicate with stakeholders. Previous works on BSC in health care^1^, ^7^, ^8^, ^9^, ^12^, ^15^, ^27^, ^28^, ^31^, ^34^, ^35^ deliver similar conclusions.

When BSC is used as a monitoring system it promotes a new and shared language inside the organization and in the health care network.^1^ As our findings highlight, the top managers' accountability behaviour increases.^9^, ^35^ The activity of monitoring hospital results,^8^, ^9^, ^34^ strategic units' performance,^12^ ward activities and behaviours,^15^ and patient satisfaction^28^ still remains an important function of BSC. In BSC potentials, this should be the opportunity to install an external viable communication with the administration of the hospital and the government.^1^ Moreover, the monitoring activity could find an interesting expected use in terms of benchmarking results across hospitals.^27^, ^31^ All the hospitals and their health care providers could be monitored, and the comparison suggests specific areas of concern.^34^

### 3.3 Strategic decision‐making use of BSC

The IUH analysis does not suggest a decision‐making use of BSC.^10^ In 2013, perspectives and measures of BSC monitor all the strategic objectives and the process of introduction facilitates the attention focussing on core activities and priorities. In 2017, the strategic decision‐making process seemed manipulated by external forces. This fact was evident when the region adopted two measures. First, it decided a different allocation of radiopharmaceuticals management, and second, it prevented IUH from buying a linear accelerator. Frequent inspections, mandated information systems implementation, and data requirements support this interpretation.

In general, extant studies reveal minor strategic decision‐making use of BSC.^14^, ^19^, ^20^ Peters et al.^34^ report an improvement of decision‐making processes among all the actors that participate in the design process. Inamdar and Kaplan^13^ and Gordon^12^ describe voluntary initiatives of BSC implementation in which measures give executives and strategic units' managers an effective tool for decision‐making and strategy implementation.^25^, ^46^ Finally, two studies mention this type of use in terms of expected use.^5^

The IUH results signal a further point of attention. The top managers in 2013 and 2017 seem to have a completely different style of leadership. Modell's^21^ findings illustrate the importance of leadership styles according to the strategy definition. According to Modell,^21^ our results shed light on how top managers (i.e., change agents) and external forces (i.e., political, social and economic realities) affect the strategy discourse. In 2017, top managers seem to have a propensity to ally themselves with politically elected bodies.^3^ This propensity narrows the scope of strategic objectives and increases compliance with regulatory pressures. In fact, the current top management adopts for BSC the same indicators required by the region and does not resist the external directions.

As the interviewees observed, the top managers in 2013 set clear priorities. They were aware that a large amount of information negatively affects the decision‐making process and the execution of strategy. Thus, they prioritized objectives according to political and organizational priorities. The Medical Director and the General Manager took the opportunity of the spending review to promote an innovative reorganization of internal processes. They forced all the organizational members to focus on their priorities and they obtained political endorsement. Finally, the 2017 situation shows the hospital fully committed to compiling all the information required by the external public organizations. They compile more information than is required for decision‐making.^3^, ^40^ BSC objectives seem to have a strictly short‐term emphasis and it appears to be a mandated system as well one that deals with the annual budget, regional information flows, interregional performance benchmarking and so on. According to this view, the current top managers attribute to BSC the main role of legitimacy provider.

### 3.4 Use of BSC for legitimation

Using BSC for legitimation means providing justification for actions or decisions made in the past.^10^ In the IUH, as the Management Accounting Manager mentioned, the top managers prepare themselves 1 week before the inspection takes place. They want a defence against the accusation. During this preparatory phase, top managers and staff managers use BSC to collect data.

Except for one case,^4^ previous studies do not attribute to BSC the role of legitimizing the system. This appears curious to us as the performance measurement myths in the public sector mention the consensus‐building activities profusely.^3^ Indeed, the Northcott and France^4^ mention the general process of implementation, speculating an expected use of BSC that provides a result similar to our conclusion. Precisely, they argue that ‘while it [BSC] may serve an interim purpose as a symbolic shield against accusations of poor or outdated performance management’ (p. 44), it cannot be presumed that BSC easily pervades management practices and decision‐making.

Starting from our case study, literature on public health domains could benefit from in‐depth descriptions of the four types of use stated by Henri.^10^ In particular, cases in which BSC supports strategic decision‐making and legitimizing purposes could reveal interesting coupling and de‐coupling situations.^3^, ^15^, ^21^ Further, adopting a holistic view of BSC design‐implementation and use stimulates the emergence of political, social and economic realities. These forces affect the use of the system and the strategy dialogue in the medium to long term and reveal the potential of BSC as a strategic management tool.^19^, ^20^

## 4 CONCLUDING REMARKS

In the lack of studies that consider the dynamism of BSC, the contribution of this paper is threefold. First, the study demonstrates that the use of BSC changes over time. Recently, Bobe et al.^16^ found a different implementation of BSC in a healthcare organization compared with the design at the system level. No previous studies reveal different uses of BSC in the same organization. We label this result ‘dynamic use’, recognizable in longitudinal explorations. The IUH case study provides the first results of dynamic and legitimizing use of BSC. IUH is embedded in the Italian context. No previous literature taken into consideration in this study run in a similar context.^2^ Contrary to other countries, such as United States, Sweden, Canada and New Zealand,^4^ in Italy, no BSC mandatory initiative was developed with the exception of the Decree Law no.150/2009, which strongly stressed the introduction of PMSs in public organizations. This decree does not force top managers to implement the framework of BSC. However, this study shows that BSC through its implementation and evolution can be a valuable tool for pursuing organizational legitimacy. Second, the paper proposes a theoretical contribution, adopting the Henri's^10^ types of PMS use discloses an interesting practice. BSC could provide justification of actions and decisions (i.e., legitimization use of BSC) as well as monitoring results, supporting strategic decision‐making processes, and focussing attention on priorities. Third, the paper proposes a novel point on BSC as a pivotal lever in a PMS that can be governed by the managers as a strategic management tool for facing legitimacy pressures.

Being explorative in its nature, the study has some limitations. First, we refer our empirical investigation and conclusions to the Henri's^10^ model that encompasses four uses of PMSs. Other plausible uses might have been investigated and they might have provided interesting results. Second, we interviewed the key stakeholders directly involved in the BSC design and implementation over the years. Excepting the evaluation committee member, they are all organizational actors. External political and administrative stakeholders might have given other interpretations regarding the use of BSC. Third, considering differences in the design and use of BSC among private and public organizations depending on their size and institutional context, results may not be generalized outside the scope of one current sample (i.e., 500‐bed hospital in Italy).

Future studies will consider the BSC dynamic use comparing different countries and organizations both from the public and private sector and supporting the BSC evolution through the employment of the institutional and legitimacy theories. In addition, further efforts should be considered in exploring the opinion of a wider range of stakeholders involved in the BSC design but also influenced by the BSC application.

## ETHICS STATEMENT

Not applicable.

## Data Availability

Data available on request due to privacy/ethical restrictions. The data that support the findings of this study are available on request from the corresponding author. The data are not publicly available due to privacy or ethical restrictions.
